# Modeling Social Dominance: Elo-Ratings, Prior History, and the Intensity of Aggression

**DOI:** 10.1007/s10764-017-9952-2

**Published:** 2017-03-16

**Authors:** Nicholas E. Newton-Fisher

**Affiliations:** Living Primates Research Group, School of Anthropology and Conservation, University of Kent, Canterbury, Kent CT2 7NR UK

**Keywords:** Budongo, Chimpanzee, Hierarchy, *Pan troglodytes*, Rank

## Abstract

Among studies of social species, it is common practice to rank individuals using dyadic social dominance relationships. The Elo-rating method for achieving this is powerful and increasingly popular, particularly among studies of nonhuman primates, but suffers from two deficiencies that hamper its usefulness: an initial burn-in period during which the model is unreliable and an assumption that all win–loss interactions are equivalent in their influence on rank trajectories. Here, I present R code that addresses these deficiencies by incorporating two modifications to a previously published function, testing this with data from a 9-mo observational study of social interactions among wild male chimpanzees (*Pan troglodytes*) in Uganda. I found that, unmodified, the R function failed to resolve a hierarchy, with the burn-in period spanning much of the study. Using the modified function, I incorporated both prior knowledge of dominance ranks and varying intensities of aggression. This effectively eliminated the burn-in period, generating rank trajectories that were consistent with the direction of pant-grunt vocalizations (an unambiguous demonstration of subordinacy) and field observations, as well as showing a clear relationship between rank and mating success. This function is likely to be particularly useful in studies that are short relative to the frequency of aggressive interactions, for longer-term data sets disrupted by periods of lower quality or missing data, and for projects investigating the relative importance of differing behaviors in driving changes in social dominance. This study highlights the need for caution when using Elo-ratings to model social dominance in nonhuman primates and other species.

**Electronic supplementary material:**

The online version of this article (doi:10.1007/s10764-017-9952-2) contains supplementary material, which is available to authorized users.

## Introduction

Social dominance is a widespread phenomenon across multiple taxa. Strictly, it is a property of dyadic relationships, i.e., a summation of agonistic interactions between particular and specific pairs of individuals (Drews 1993; Hinde 1976). Classically, social dominance has been determined by analysis of the outcome of fights (Bernstein 1981; Drews 1993), although it is common for this to be extended to include a variety of agonistic interactions in which the outcome is clear (where one individual clearly loses), with a persistent winner recognized as dominant to the other member of the dyad (Briffa *et al*. 2013). Where animals form social groups, it is possible to determine an ordering of individuals from their dyadic social dominance relationships: the dominance hierarchy. Such hierarchies are an element of group structure (Hinde 1976), often persisting despite shifting group memberships and changes in the identities of individuals that occupy particular ranks.

A variety of analytical techniques have been employed to derive dominance hierarchies and individuals’ ranks within these from agonistic interaction data (de Vries 1998; Whitehead 2008). Now-traditional methods, including Clutton-Brock’s index (Clutton-Brock *et al*. 1979), the I and SI method (de Vries 1998; Schmid and de Vries 2013), and David’s scores (David 1987), consolidate interactions observed over a particular time period to determine a single ranking for that period. Although such methods have been widely used, and are useful in recovering linear hierarchies if these exist in the data, they suffer from a particular shortcoming in that they necessarily obscure any variation in hierarchy position during the period for which observations are consolidated (often a number of months, or a year), with the consequent effect that use of derived rankings in analysis of social behavior, e.g., grooming, mating, at particular points within that period may not accurately reflect the rankings of individuals at the time of the interactions in question.

By contrast, the Elo-rating method (Albers and de Vries 2001; Elo 1978) for modeling dominance hierarchies allows for an ongoing determination of rank. This method tracks rank trajectories as a consequence of wins and losses in encounters with other individuals. Individuals are ascribed probabilistic point estimates (Elo-ratings) of their competitive abilities, with numerically greater ratings indicating more successful competitors. Elo-ratings are interval-level and thus provide a cardinal ranking of individuals. While an individual’s Elo-rating does not depend directly on those of others—this can change without affecting those of at least some of its other competitors, and an individual’s relative position (ordinal ranking) might change despite an unchanging rating—its value is the result of a history of competitive interactions. It is meaningful therefore only in comparison with that of others, whether these form a baseline against which it can be judged or because past and future shifts in Elo-ratings depend on the relative ratings of contest opponents. Individuals with similar Elo-ratings (and thus competitive abilities) may be considered to belong to the same “category” or “class” (Elo 1978), within which individuals have inconsistent or undecided dominance relationships, while dissimilar Elo-ratings are predictive of clear dyadic dominance relationships. The size of each class (Elo’s “class interval,” the spread of Elo-ratings within a class) is likely to differ between species, and may vary within a single hierarchy. For nonhuman primates, such class intervals remain to be determined empirically: Elo’s (1978) choice of 200 was an arbitrary reflection of tradition among human chess players.

Since the publication of appropriate R functions by Neumann *et al*. (2011), use of Elo-ratings to model dominance hierarchies and to derive ranks for additional analyses has blossomed, such that this may now be the default method within studies of nonhuman primates (Cassalette *et al.* 2016; da Silva *et al.* 2016; Fedurek *et al.* 2015a; Franz *et al.* 2015; Kaburu 2013; Kaburu and Newton-Fisher 2013, 2015a; Murray *et al.* 2016; Pusey *et al.* 2016; Schoof *et al*. 2016; Spillmann *et al*. 2016; Wooddell *et al*. 2016; Young *et al.* 2016). Two deficiencies exist within this method, however, that can compromise any subsequent analysis conducted with derived ranks or extracted Elo-ratings. The first of these deficiencies is the method’s approach of starting all individuals at the same Elo-rating, updating this as interactions are added across the observation period. The second is the explicit assumption that all interactions entered into the model are equivalent in their potential influence on rank trajectories, with this mediated only by the respective ranks, i.e., Elo-ratings, of the two participants before their interactions. I discuss each of these in the text that follows.

### Initial Elo-Rating and Prior History

In the absence of any knowledge of the dominance relationships within a group of animals, the method of calculating Elo-ratings assumes no differences between individuals, essentially that dominance relationships are unresolved, and assigns equal Elo-ratings (and thus equal ranks) to all group members. Although this may be appropriate if a group is newly assembled, in many species where sociality is persistent (numerous mammalian orders, for instance) this assumption is unwarranted and will introduce error into any attempt to model social dominance hierarchies, individual ranks (whether ordinal or cardinal), or rank trajectories. Traditional methods of assessing social dominance have dealt with this by accumulating data over a sufficiently long period to resolve dominance relationships, but this approach carries with it an implicit and untested assumption that social dominance relationships are stable over the period of accumulation.

For the Elo-rating approach that explicitly tracks hierarchy position and rank trajectories, a period of time sometimes referred to as the “burn-in” period is necessary for sufficient observations to accumulate for the modeled rankings of individuals to catch up with real dominance relationships (Albers and de Vries 2001; Neumann *et al.* 2011). During this period, Elo-ratings and derived ranks are unreliable, as the Elo-ratings attached to individual animals are not yet reflective of their relative position in the hierarchy. Ratings and ranks derived during this period should, therefore, be disregarded and not used in subsequent analysis. The extent to which this is a problem for any particular study depends on an interaction between the rate of dominance-related interactions, sampling intensity, and the duration of the study period. Both Albers and de Vries (2001) and Neumann *et al*. (2011) are, however, somewhat vague about precisely how long this might be, probably not least because the duration of this period will vary with the frequency of agonistic interactions in any particular organism. For a species in which individuals interact frequently, and a social group is observed over a long period of time, this burn-in period will be short, and discarding both ratings and ranks from this period will have little impact on the study. By contrast, for a species that interacts at a relatively slow rate, or where sampling of such interactions results in an equivalently small number of observations, the burn-in period may represent much or all of the observation period, making it difficult or impossible to use Elo-ratings to rank individuals reliably.

Clearly, in the absence of other information, individuals either must be considered to be equal or be assigned starting values at random. However, in many cases subjects may have been observed before, during previous research or a pilot study. Where some knowledge of the dominance hierarchy exists prior to a particular study period, including this information should reduce the duration of the burn-in period. For studies conducted in discrete field seasons, ranks and hierarchies may shift while the animals are not observed; for a PhD student conducting fieldwork, others may have collected data previously; in long-term data sets, observations may be interrupted by extrinsic factors ranging from civil disturbance to varying staffing levels, or periods of observation might have data of questionable quality. In each of these, and it is possible to conceive of additional examples, the data with regard to dominance ranks can be considered disrupted: some prior knowledge of rank (in the general sense of hierarchy position) exists, but is either of lower quality or separated in time from the period of high-quality data to which the Elo-rating method is to be applied. A method of using that prior knowledge would, therefore, be particularly helpful if it reduced or eliminated the burn-in phase.

### Equivalence of Interactions and the Intensity of Aggression

In many species, dominance is expressed, and potentially reinforced, through relatively low levels of aggression. Typically, such behavior by the dominant is described as a threat, and supplants (or displacements) also fall into this category. The subordinate individual responds appropriately, and the contest does not escalate. When researchers record observations of interactions, such behavior provides a marker of dominance relationships. Primatologists have conventionally made use of approach/retreat encounters to identify winners and losers, and so rank individuals (Cowlishaw and Dunbar 1991), but such data may be supplemented or even replaced by aggressive interactions across a variety of types and intensities (Engelhardt *et al*. 2006; Muller 2002; Newton-Fisher 2002b; Silk 1993; Tiddi *et al*. 2012), as is done in studies of diverse other taxa, e.g., cichlid fish (*Metriaclima zebra*: Chase *et al*. 2002), red deer (*Cervus elaphus*: Clutton-Brock *et al.* 1979), crayfish (*Astacus astacus*: Goessmann *et al*. 2000), and wolves (*Canis lupus*: Sands and Creel 2004). Accumulating a set of such interactions is typical across studies, regardless of the method employed to determine ranks.

In the Elo-rating method, the variable *K* is used to determine the degree to which each interaction influences the future rank trajectory of both winner and loser. When introducing Elo-ratings to behavioral ecology, both Albers and de Vries (2001) and Neumann *et al*. (2011) chose to hold this variable constant. This was not particularly at odds with previous practice, but holding *K* constant highlights the assumption implicit in other methods that, so long as a clear winner and loser can be identified, variation in the intensity of aggression does not influence social dominance rank (hierarchy position) or rank trajectories: all behaviors within an interaction are weighted equally in this determination, with only the outcome being considered important. In some studies in which intensity of aggression is considered, only a subset of agonistic interactions (typically those of greater intensity) are used in determining rank (e.g., Muller 2002): this carries an implicit assumption that intensities above the cut-off are equivalent in this determination, while those falling below do not contribute. It is not obvious *a priori* where such a cut-off should occur, however, if indeed it should at all. Interactions involving low-intensity aggression may only serve to reinforce current ranks, or they may contribute to future rank trajectories if participants take winner and loser roles as a consequence. If they do contribute to future rank trajectory, it seems unlikely that their impact will be as great as that of high-intensity interactions. Thus it would seem sensible to include all win–loss interactions in a determination of Elo-ratings, but allow their relative contributions to vary. This can be achieved by adjusting the value of *K* accordingly (Albers and de Vries 2001; Franz *et al.* 2015; Neumann *et al.* 2011). Allowing *K* to vary makes assumptions about the impact of differing levels of intensity explicit, allowing hypotheses, e.g., regarding the aforementioned cut-off, to be modeled.

Here, I present two developments of Neumann *et al.’s* (2011) R functions to address these deficiencies and so extend the usefulness of using Elo-ratings to model the dominance hierarchies in animal groups: 1) incorporation of prior history of dominance ranks (hierarchy positions); and 2) the explicit recognition of differing intensities of aggression in agonistic interactions. I demonstrate this code using behavioral observations of wild male chimpanzees (*Pan troglodytes*). This is a useful species with which to test these extensions: male chimpanzees demonstrate a wide variety of aggressive behaviors that scale in intensity, while many groups have been studied repeatedly but with breaks or variation in data quality through time, such that prior histories of social dominance exist, but with omissions or lack of detail. Further, chimpanzees display a highly characteristic and socially salient vocalization, the “pant-grunt,” by which subordinates demonstrate and acknowledge their status relative to recipients (Bygott 1979; Goodall 1986; Newton-Fisher 2004; Noë *et al.* 1980). While closely matched individuals rarely pant-grunt to one another and those contesting rank may withhold pant-grunts to maintain ambiguity about their dominance relationship (Hayaki *et al*. 1989; Newton-Fisher 2004), when one adult male pant-grunts to another, this marks a clear recognition of a subordinate–dominant relationship.

Validating rankings derived from observed interactions is difficult, given that these should already represent the best effort to model an underlying and otherwise inaccessible real hierarchy. A second interaction type with an unambiguous relationship to dominance rank is necessary in order to compare hierarchies constructed under different assumptions. For chimpanzees, the directionality of pant-grunt vocalizations provides such a measure. If an Elo-rating model of the hierarchy is accurate, individuals with decided dominance relationships should have quite distinct Elo-ratings, i.e., not be in the same “class”, with the dominant member of a dyad having a higher rating. By contrast, those with undecided or inconsistent dominance relationships should have similar Elo-ratings (Elo 1978), as noted previously. Therefore, pant-grunts directed from high-rated individuals to low-rated individuals indicate errors in the calculated Elo-ratings, while those between individuals with similar ratings demonstrate—by definition—that these individuals are not in the same “class”. In consequence, a record of pant-grunt interactions (or equivalent subordinate behavior in other species) provides a minimum standard against which hierarchies derived from agonistic interactions can be judged.

In this study, I construct multiple Elo-rating hierarchies to demonstrate the impact of incorporating prior history (both through ordinal ranks and rank categories such as high, medium, low), and varying intensities of aggressive interactions. I determine the accuracy of these hierarchies by comparing Elo-ratings to the record of pant-grunting, and show how incorporating prior history and interaction intensity influences the interpretation of rank effects on other social interactions by looking at the relationship between rank and male mating success.

## Methods

### Data Collection

I collected data on agonistic interactions between October 2003 and August 2004 (1265 h of observations) from chimpanzees of the Sonso community in the Budongo Forest Reserve, Uganda. This community inhabits *ca*. 7 km^2^ (Newton-Fisher 2002a) of the 428-km^2^ semideciduous tropical forest within the reserve (Eggeling 1947; Plumptre 1996; Reynolds 2005), and has been studied continuously since 1994 (Newton-Fisher 1997; Reynolds 2005). During data collection, the community consisted of 63 individuals including 8 adult males (≥16 yr old), 6 adolescent males (ranging from 9 to 14 yr old), and 21 adult females (≥14 yr old).

Owing to the fluid (fission–fusion) nature of the chimpanzee social system, whereby individuals associate in small subgroups (parties: Sugiyama 1968), I recorded data using a focal-behavior sampling regime. This was equivalent to an all-occurrence method within the party of a focal individual. A focal individual was necessary in order to determine which chimpanzees to follow and observe when parties fragmented, and I selected focal individuals from a list of six adult males and six adult females. I attempted to alternate between male and female focal subjects across days, and to select individuals that were less followed than others on the list. This was not always possible because of the fluid social system, but I left parties with heavily followed individuals to search for others. I recorded identities of the aggressor and the target of aggression, details of the behavioral interaction, and its outcome. I recorded the full sequence of interactions that escalated or included multiple aggressors, e.g., retaliations and the engagement of third parties. I also recorded a variety of contextual data such as party composition and other forms of social and sexual interaction (including a full record of the number of copulations). I recorded data using pen-and-paper, and by audio narration into a Sony MiniDisc recorder.

To determine social dominance and hierarchy rank, I identified the winner of each interaction based on the outcome, and specifically on the behavior of the individual judged to have lost (through demonstration of behaviors such as screaming, cowering, or running away, or by receiving but not returning physical violence and/or wounds). I considered aggressive behaviors that provoked no response from the target to be ineffective in influencing social dominance relationships and discarded these from the analysis. I accorded a measure of intensity to each aggressive interaction according to the scheme in Table I, itself an elaboration of the intensity scale presented by Goodall (1986). This scheme accords increasing intensity to multimodal threats and to those that involve approaching the target; the highest levels of intensity relate to assault where the target is struck once, or multiple times, and whether wounding occurs. For the purposes of these analyses, I distinguished among *static threats*, *approach threats*, *undirected charging displays* (those without a clear target, but where an individual responded as if targeted), *directed charging displays* (where these were targeted at a particular individual), *chases* (with no contact), and *attacks* (Goodall 1986). I assigned a different *K* value to each of these categories, using the default value of 200 (Neumann *et al.* 2011) for the most commonly observed form of aggression (the *directed charging display*), and scaling up and down in multiples of 25 to distinguish varying intensities (Table I). Although this approach was somewhat arbitrary, it represents a first step in quantifying variation in intensity, given that the suggestions for objectively determining *K* values provided by Albers and de Vries (2001) are impractical where dominance relationships are already established. Where a single interaction escalated through a number of intensities, I classified the interaction according to the most intense level of aggression displayed by the winner.

**Table I:** Intensity of aggression as shown by male chimpanzees (*Pan troglodytes*) together with associated *K* values as assigned in this study and the number of interactions at that intensity

Intensity scale	Categorization of aggression	*K*	No. of decided interactions
1	Threat/static/*vocalise*	$$ \left.\begin{array}{l}50\hfill \\ {}50\hfill \\ {}50\hfill \\ {}50\hfill \end{array}\right\} $$	38
2	Threat/static/*gesture*		
3	Threat/static/*gesture & vocalise*		
4	Threat/static/*use object*		
5	Threat/approach/*vocalise*	$$ \left.\begin{array}{l}100\hfill \\ {}100\hfill \\ {}100\hfill \\ {}100\hfill \end{array}\right\} $$	53
6	Threat/approach/*gesture*		
7	Threat/approach/*gesture & vocalise*		
8	Threat/approach/*object*		
9	Charging display/no target	150	5
10	Charging display/through party	175	22
11	Charging display/targeted	200	125
12	Chase/no contact	225	97
13	Attack/strike in passing	250	26
14	Attack/< 30s duration	300	35
15	Attack/> 30s &/or serious injury	375	4
16	Attack/> 5min duration &/or fatal	*–*	*not observed*

Larger *K* values result in greater influence on the Elo-ratings of both winners and losers following an interaction. Italicized descriptors were not differentiated by *K* value. The four levels of Attack correspond to those distinguished by Goodall (1986)

To examine the importance of using prior history of dominance relationships, I used data collected from the same community before 2003 to determine an ordinal ranking of male chimpanzees at the start of the 2003–2004 data collection period. I conducted the first study of social dominance in this community in 1994/1995 (Newton-Fisher 1997, 2004), and to determine changes in male rank from the 1994/1995 values, I consulted long-term records (*cf*. Newton-Fisher *et al.* 2010) collected under direction of V. Reynolds, as well as the reports of research projects conducted between 1995 and 2003 (Arnold and Whiten 2001; Fawcett 2000; Notman 2003; O’Hara 2005; Oliver 2002). To show how incorporating prior history and interaction intensity influences the interpretation of rank effects on other social interactions, I tallied the number of copulations (*N* = 653) achieved by each male and examined the relationship between this variable and rank (as determined by the Elo-rating hierarchies).

To determine the accuracy of the hierarchies generated by the Elo-rating function, I compared daily ranks from each hierarchy to observed pant-grunt interactions, as this vocalization is considered to be an unambiguous indicator of the direction of the subordinate–dominant relationship (Bygott 1979; Goodall 1986; Hayaki *et al.* 1989). My data set contained 1001 pant-grunts exchanges within 66 (of a possible 91) male dyads. While 9 of the 14 males were recipients, a single male (DN) was the recipient in more than half (539/1001) of these interactions. My purpose with this comparison was not to find a hierarchy that predicted a high percentage of pant-grunt interactions, but to identify categorical errors: given a choice of hierarchies derived from the same data under different assumptions, the preferred hierarchy is that which minimizes, and preferably eliminates, such errors. These errors were the inconsistencies between directionality of pant-grunting and relative hierarchy position, as relative ranks in the hierarchy are predictive of dyadic dominance relationships (except for individuals within the same “class” that by definition have undecided dominance relationships and so should not exchange pant-grunts).

### Data Analysis

I edited the R function *elo.sequence* written by Neumann *et al*. (2011). As far as possible, I preserved the code largely as written, and attribute full credit to Neumann *et al*. for their work. My edits implement two extensions to their code: 1) to accept an initial ranking of individuals—that is, social dominance rankings prior to the period of data to be analyzed; and 2) to allow their constant *K* to become a variable such that each interaction can be assigned a particular value. These values are provided by the user in the data file called by the *elo.sequence* function:

         elo.sequence(datafile = "c:\\example data.csv", sep = ",", startingvalue = 1000, constant_k = 200, priorElo = list(), priorRanks = list(), priorRankCategory = list(), priorRankIndex = 0, outcome = 1)

**Fig. 1:**
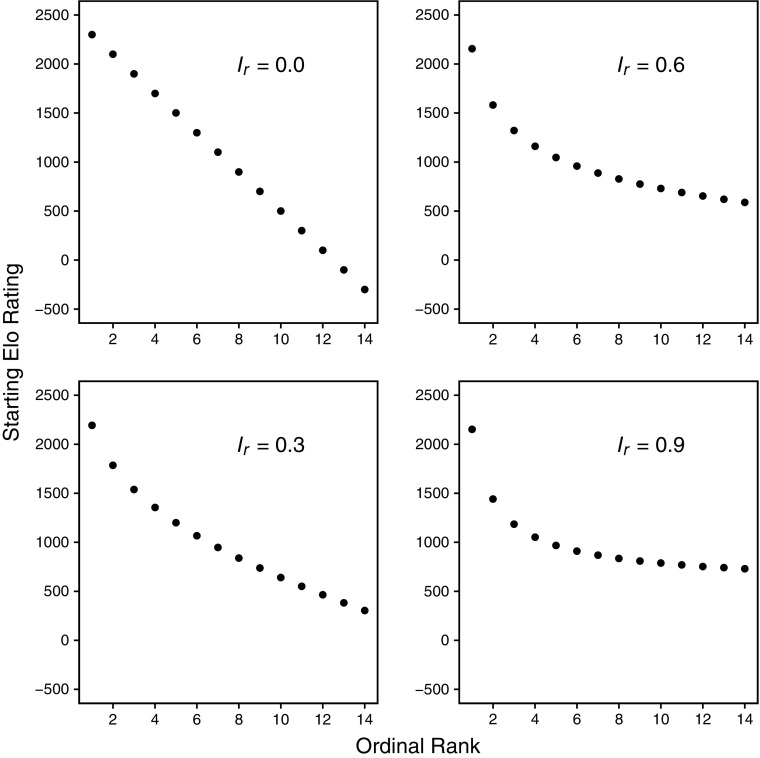
Impact of the *elo.sequence* function argument *I*_*r*_ (the “priorRankIndex”) on starting Elo-ratings generated by this function from user-supplied prior history of dominance interactions in the form of an ordinal ranking of individuals.

This modified version of *elo.sequence* accepts four additional input variables, or arguments. Three of these are case-sensitive lists of subjects and associated rankings derived from previous study (the prior history): 1) priorElo: subjects and Elo-ratings in the form (id1 = 1500, id2 = 1200, …); 2) priorRanks: subjects and ordinal ranks in the form (id1 = 1, id2 = 2, …); and 3) priorRankCategory: subjects and ordered categorical ranks in the form (id1 = alpha, id2 = high, id3 = medium, id4 = low). The priorRankCategory list can be incomplete, e.g., only identifying an alpha male, or high vs. low, with unlisted subjects initially assigned the Elo-rating specified by the startingvalue argument. These three arguments are processed in turn, so that user-provided Elo-ratings are used in preference to a user-supplied ordinal, or ordered categorical, rankings. Where either an ordinal or categorical ranking is provided, the initial Elo-rating (*E*) for each individual *i* is calculated according to this equation:

$$ {E}_i={S}_e+\left[\left({x}_r-{S}_{r i}\right)\times K\times {S}_{r i}^{-{I}_r}\right] $$

where *S*_*e*_ is the starting Elo-rating supplied to the function call (default = 1000); *x*_*r*_ is the median of the supplied ranks derived from prior history; *S*_*ri*_ is the starting ordinal rank for individual *i*; *K* is the user-supplied “constant_k” value (default = 200); and *I*_*r*_ is the user-supplied priorRankIndex (default = 0), which specifies a nonlinear (reciprocal power) relationship between the user-supplied ranking and starting Elo-ratings. Larger values of *I*_*r*_ result in a relatively greater starting Elo-rating for the highest ranked individual (ordinal rank 1) while Elo-ratings for low-ranking individuals become more compressed (Fig. 1). Where prior history is in the form of ordered categorical ranks, individuals within each category are assigned appropriate ordinal ranks for the purpose of determined starting Elo-ratings: alpha = 1; high = *N*/4; medium = *N*/2; and low = *N – N*/4, where *N* is the total number of subjects in the analysis. This creates an even spread of the four categories (but not necessarily of individuals), which can be modified by adjusting the value of *I*_*r*_ as described previously. Calculated Elo-ratings are centered on the supplied starting value, *S*_*e*_.

If these arguments are not specified, (i.e., are allowed to take their default values, which for the lists is *null*) the *elo.sequence* function reverts to the behavior described by Neumann *et al*. (2011). I also modified the code to provide the user with the option of specifying an existing R dataframe instead of an external data file. If a data file is specified, however, it must be in CSV format rather that in Microsoft Excel®^’^s native format (this modification—which improves compatibility across operating systems—is courtesy of Christof Neumann). The layout of the data file is shown in Table II, while the R script for *elo.sequence* is provided in the Appendix [ESM2]. Note that this function should be used alongside the other functions (*elo.extract*, *elo.plot*, *elo.single*) provided by Neumann *et al*. (2011).

**Table II:** Datafile header for the R function *elo.sequence*, modified from Neumann et al. (2011) to accommodate varying *K* values

Date	Time	Winner	Loser	*K*	Outcome
2003-08-10	15:34	ZF	TK	100	1
2003-10-13	08:56	DN	ZF	200	1
2003-10-16	08:44	ZF	TK	200	1
2003-10-24	09:11	ZF	MA	275	1
2003-10-28	11:09	ZF	NK	200	1
2003-10-28	11:11	DN	ZF	200	1
2003-10-28	11:12	ZF	TK	200	1
2003-10-28	12:05	NK	TK	200	1
2003-10-28	12:36	NK	TK	100	1

If *K* is left blank across all observations in the datafile, *elo.sequence* will use the value provided in the function call for all interactions (default = 200)

For each aggressive interaction between adult or adolescent males, I assigned a level of intensity and the associated *K* value (Table I). To determine the starting ranks (*S*_*ri*_), I ranked all individuals using the conventional approach in which the most dominant individual (the alpha male) is ranked as 1, using data from previous studies (see earlier). I assigned ordinal ranks in descending age order for adolescent males that were otherwise without a prior rank. I also assigned individuals to ordered categorical ranks (alpha, high, medium and low: *cf*. Bygott 1979) to explore the effect of using a prior history that offered only limited resolution of a dominance hierarchy. I used the modified *elo.sequence* function in R v2.14.2, and latterly v3.3.0 (R Core Team 2016), to generate hierarchies for the Sonso chimpanzees for the period between December 2003 and August 2004, and I used *elo.extract* (Neumann *et al.* 2011) to determine Elo-ratings and ordinal ranks for each day for which I had recorded observations of either pant-grunt vocalizations or successful copulations. I generated six hierarchies: a) without employing my extensions to the function; b) applying prior history in the form of ordinal ranks, with a linear relationship between starting Elo-ratings and rank; c) applying prior history (ordinal ranks), with an *I*_*r*_ value of 0.3; d) applying prior history (ordinal ranks), an *I*_*r*_ value of 0.3, and intensity of aggression (variable *K*); e) applying prior history in the form of ordered categorical ranks, an *I*_*r*_ value of 0.3, and intensity of aggression; and f) applying very limited prior history distinguishing only an alpha male, an *I*_*r*_ value of 0.3, and intensity of aggression. I chose the value of 0.3 after examining the effect of a range of values (0.1–0.9) on the ${S}_{r i}^{-{I}_r}$ term prior to generating the hierarchies. This was sufficient to stretch the highest ranking individual away from the rest without imposing any extreme pattern: altering *I*_*r*_ affects the steepness of the hierarchy from which starting Elo-ratings are generated, with larger values resulting in smaller average interindividual differences in Elo-ratings, and thus more egalitarian hierarchies (de Vries *et al*. 2006). At the same time, however, larger *I*_*r*_ values also increase the distance between the alpha individual and others, which may correspond to despotism, and future work should explore the possibility of deriving the most appropriate values from measures of hierarchy steepness (de Vries *et al.* 2006).

I examined mating success in light of both the default (a) and modified (d) modeled hierarchies. I determined Spearman rank correlations between ordinal rank and number of copulations achieved using rank data from each of the two models, and Pearson correlations between the mean rank for each male (using both ordinal rank and Elo-rating, determined for each day on which the male copulated) across the data set and number of copulations achieved. Mean ranks, and number of copulations, were normally distributed.

## Ethical Note

This research complied with regulations set by the Ethics Committee of the University of Kent, the protocols of the Budongo Forest Project (now BCFS), and the legal requirements of Uganda.

## Results

I identified 405 aggressive interactions between adult and adolescent male chimpanzees in which a clear winner and loser could be determined. Of these, 91 had a maximum intensity of *threat* (38 static, 53 approach), 27 of *undirected charging display*, and 125 of *directed charging display*. A further 97 involved a *chase* but not physical violence, while 68 culminated in an *attack*: 26 in which the target was *struck in passing*, and 35 in which an assault of <30 s duration was directed at the target. Only 4 attacks were prolonged and resulted in obvious wounding. I observed no fatal attacks among adult and adolescent males during this period.

**Fig. 2:**
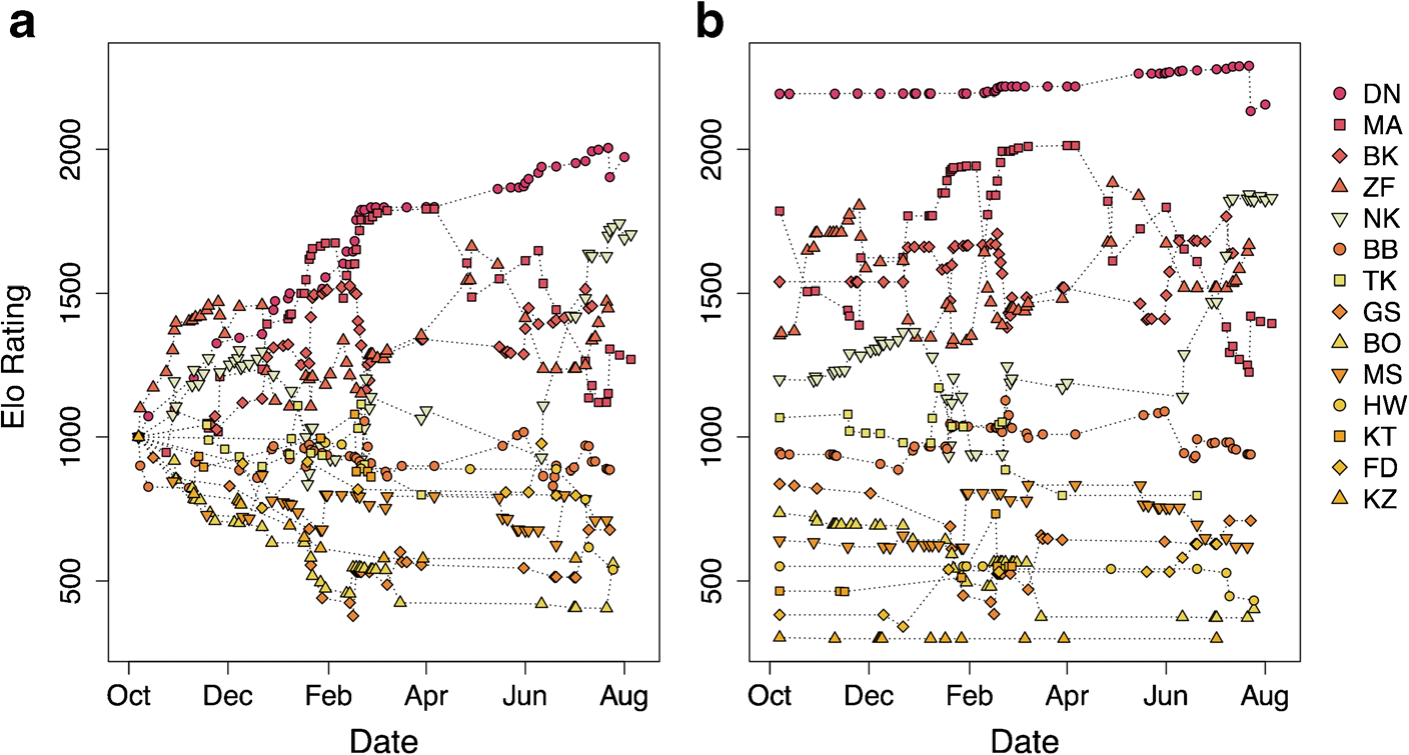
Rank trajectories for the adult and adolescent male chimpanzees (*Pan troglodytes*) of the Sonso community (Budongo Forest, Uganda) between October 2003 and August 2004, as determined by an Elo-rating model **(a)** following the default parameters proposed by Albers and de Vries (2001) and Neumann *et al*. (2011); **(b)** assigning starting Elo-ratings according to prior records of dominance ranks, applied using a negative exponential, and allowing impact of interactions to vary depending on intensity of aggression.

I found that with these data, the standard Elo-rating process (Albers and de Vries 2001; Neumann *et al.* 2011) failed to resolve a male hierarchy (Fig. 2a) and generated 14 inconsistencies with the pant-grunt data (Table III): each inconsistency was an occurrence of the model predicting an individual to be the dominant member of a dyad when the pant-grunt data showed the reverse, including the identity of the alpha male. Four of these inconsistencies were of the putative alpha male pant-grunting to another adult male. In only one of these inconsistencies were Elo-ratings particularly close, differing by only 9 points. Given the social salience of these vocalizations, such inconsistencies are serious errors.

**Table III:** Inconsistencies between rankings derived by using Elo-rating under different assumptions and the directionality of pant-grunt vocalisations, using data from male chimpanzees (*Pan troglodytes*) of the Sonso community, Budongo Forest, Uganda, collected between October 2003 and August 2004

Model	Analysis	No. of inconsistencies (no. of dyads)	Difference in Elo-rating (mean ± SD)
a	Unmodified (Neumann *et al.* 2011)	14 (9)	154.64 ± 103.60
b	Prior history^a^ with linear relationship	2 (2)	103.00 ± 97.58
c	Prior history^a^ with negative exponential	3 (3)	93.33 ± 32.62
d, e	Prior history^b^ with negative exponential + intensity of aggression (variable *K*)	1 (1)	139.00

Difference in Elo-rating is the mean of the differences between members of dyads where ranks were inconsistent with the direction of pant-grunting

^a^Prior history of social dominance as ordinal ranks

^b^Prior history of social dominance as either ordinal (model d), or ordered categorical (model e), ranks

I further inspected ranks for each day on which I observed pant-grunts, and noted that the model placed individuals in biologically unlikely positions, such as young adolescents placed at times above older individuals in the upper middle of the hierarchy, and indicated three changes in the identity of the highest rated male. Only after 5 mo (around half the study period) did there appear to be any stability in the rankings, and even then, the model presents an alpha male continuing to rise substantially in relative dominance (Elo-rating) over the other males. At the time of this study, the alpha male had held his rank for almost 9 yr, and this rising Elo-rating therefore makes little biological sense. Thus, I found that the default behavior of Neumann *et al.’s* (2011) implementation of Elo-ratings resulted in much, if not all, of these data falling in what appears to be a burn-in period, such that Elo-ratings and derived ranks calculated would be unreliable as measures of relative male dominance.

**Fig. 3:**
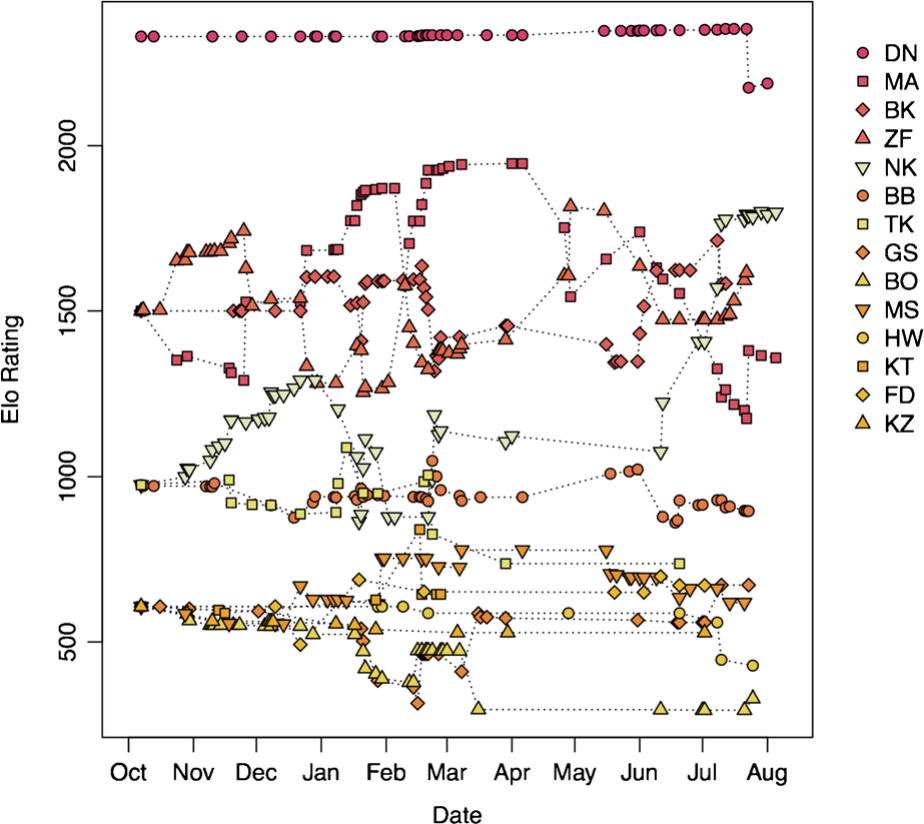
Rank trajectories for the adult and adolescent male chimpanzees (*Pan troglodytes*) of the Sonso community (Budongo Forest, Uganda) between October 2003 and August 2004, as determined by an Elo-rating model that assigns starting Elo-ratings according to prior records of ordered rank categories (alpha, high, medium, low), applied using a negative exponential, and allowing impact of interactions to vary depending on intensity of aggression.

I found that applying knowledge of prior history through ordinal ranks addressed much of this confusion, resolving most of the inconsistencies between the models and the observed pattern of pant-grunting (Table III; Electronic Supplementary Material [ESM1] Figs. S1 and S2). The model (model c; Fig. S2) that allocated starting Elo-ratings according to a power rule (a negative exponential of 0.3) rather than linearly spacing individuals (model b; Fig. S1) was more realistic for chimpanzees, with lower ranking individuals tending to cluster together in their Elo-ratings and highest ranked individuals moving apart from the rest. This model (model c) also removed the negative Elo-ratings generated by model b, although it did have a greater number of inconsistencies with the pant-grunt data (Table III). I found that applying variability in aggression intensity by means of varying *K* in addition to prior history, both through ordinal ranks (model d; Fig. 2b) and ordered categorical ranks (model e; Fig. 3), resolved all but one of the inconsistencies (Table III). This final inconsistency was between two adolescent males, one of whom was the son of a peripheral female, and who was rarely recording interacting with other males, while the other was an orphan with a (socially) dependent younger sibling that often traveled with the adult males. Thus the disparity in data between the two adolescent males (not followed as focal subjects during this study) may be responsible for the inconsistency rather than the model itself. Correctly specifying the alpha male alone removed the confusion over the highest ranked individual (by design), but left the remaining subjects to diverge from a common starting point and so did not address burn-in issues for these individuals (model f; ESM1 Fig. S3).

The apparent relationship between rank and mating success varied depending on the model used to determine rank. Under the default Elo-rating method (model a; Fig. 2a), I found only a moderate, and nonstatistically significant, correlation between extracted ordinal ranks and mating success (*r*_s_ = −0.51, df = 12, *P* = 0.060); by contrast, the correlation was much stronger (*r*_s_ = −0.930, df = 12, *P* < 0.001) when using ordinal ranks from the model with prior history and varying *K* (model d; Fig. 2b). I found similar results for the analyses of mean ranks: only moderate correlations between rank and mating success under the default model (ordinal ranks: *r* = −0.48, df = 12, *P* = 0.082; Elo-ratings: *r* = 0.56, df = 12, *P* = 0.038), but strong and significant correlations when using ranks determined by the modified model (ordinal ranks: *r* = −0.73, df = 12, *P* = 0.003; Elo-ratings: *r* = 0.73, df = 12, *P* = 0.003).

## Discussion

Although the Elo-rating method for modeling social dominance ranks and hierarchies is increasingly popular, and has much to recommend it over more traditional aggregating matrix-based methods such as the ability to track dominance ranks day by day and thereby ensure more accurate investigations of the influence of dominance on other social interactions, current formulations have certain limitations. The most obvious of these is the burn-in period. Here, I have shown that for a species such as the chimpanzee—and by extension any with a similar frequency of aggressive interaction—the burn-in can stretch over much of a typical observation period. Data collected over a 3-mo “field season” would never emerge from the burn-in period, while that collected over 12 or 18 mo, as might be typical for PhD studies (at least in the United Kingdom), might generate reliable Elo-ratings only in the last few months of study. Although this problem may not exist for species with substantially higher agonistic rates, being aware of the burn-in period, and the need to disregard Elo-ratings from this period, remains of critical importance.

The results of this study show that the burn-in problem can be largely overcome by including information on the prior history of social dominance, particularly using a negative exponential to influence the generation of starting Elo-ratings. In this study, only two individuals (MA, ZF) changed their relative position soon after the start of the observation period, and although this might indicate that their initial ranking derived from prior history was incorrect, their subsequent behavior suggests otherwise. These two males continued to jostle for rank throughout the observation period, swapping relative positions multiple times: Elo-rating model d (Fig. 2b) suggests this happened five times, which is consistent with my field observations. These two males were often close associates, but would at intervals direct aggression toward one another, and were participants in a fight that erupted, apparently unprovoked, during a grooming bout between them and that led to both receiving multiple bite wounds.

Where prior history of social dominance is known for some but not all individuals, it may be possible to infer ordinal starting ranks using other traits known to correlate with rank. In this study, I used relative age to infer ranks for adolescent males for whom I had no prior dominance history. The usefulness of such an approach will clearly depend on the strength of the correlation, as well as the accuracy with which such traits can be measured. Alternatively, prior history may be in the form of ordered rank categories, but not precise ordinal ranks. These are also useful in addressing the burn-in problem, particularly when all subjects can be assigned to categories as these effectively allocate individuals to shared ordinal ranks, with those who cannot be so assigned receiving only the default starting Elo-rating. By distinguishing rank categories on the basis of prior history, much of the “work” of tracking Elo-ratings toward realistic values is done before running the analysis. In this study, the hierarchy determined using prior categorical ranks was as consistent with pant-grunt vocalizations as that with prior ordinal ranks, despite variation in subjects’ Elo-ratings between the two models. Previous work has suggested that male chimpanzee ranks cluster into status levels (Bygott 1979; Newton-Fisher 1997, 2004), at least in some communities (Foster *et al.* 2009; Kaburu and Newton-Fisher 2015a; Muller 2002). To the extent that such structuring is real (rather than a methodological artefact), this explains why ordinal rank categories were a good match for the ordinal ranks as a form of prior history in this study. Whether a similar relationship would hold for species other than chimpanzees (or indeed across communities of this species) will depend on the particular structuring of their dominance hierarchies. If a hierarchy has a strongly despotic alpha, merely defining two ordered categorical ranks (alpha vs. others) might be sufficient. However, for chimpanzees that approach was less useful: doing so in this study did remove confusion over the alpha male, but otherwise amounted to no more than dropping a subject from the analysis with the remaining individuals (of different ordered categorical ranks) all starting from a common Elo-rating.

The best course of action when no prior history of agonistic interactions exists remains less clear. Starting all individuals at same Elo-rating is probably unavoidable in this situation (the only obvious alternative would be to assign random starting Elo-ratings), which means trying to identify, and discard results from, the burn-in phase. The results presented here suggest that the end of the burn-in phase can be detected when the alpha individual achieves a stable trajectory (except in cases where the alpha is considered separately from the other individuals), although the generality of this conclusion is likely to depend on the social dynamics of the group being studied. It is probably the case, however, that identifying some degree of stability in Elo-rating trajectories will be necessary to distinguish a burn-in period from real change. Although it might be tempting to use this to test whether an individual might be an alpha, on the assumption that this individual’s trajectory will remain stable if this true but drop rapidly otherwise, it may be impossible to distinguish these patterns from real change in the alpha individual’s trajectory or relative rank if, for example, the start of data collection coincides with the turnover of the alpha individual. Given that the results of this study suggest a burn-in phase could last many months, and perhaps the entire duration of a study, it may be better to use more traditional accumulation methods such as the I and SI method (de Vries 1998; Schmid and de Vries 2013) to estimate dominance ranks in cases where no prior history is available and agonistic interactions are at relatively low frequency, rather than to draw unreliable conclusions from inaccurate Elo-ratings. Direct comparisons between the I and SI method and the Elo-rating method (or similar approaches) have been published previously (de Vries and Appleby 2000; Neumann *et al.* 2011).

The burn-in period may be less of a problem for long-term studies where data are available over multiple consecutive years. If high-quality interaction data are available from previous studies it makes more sense to use these directly, either calculating Elo-ratings across multiple studies before extracting information for the period of interest, or providing terminal Elo-ratings from a prior study for the *elo.sequence* function via the priorElo argument. This pushes the burn-in period back to earliest period for which data are available. However, apparently contiguous long-term datasets may in fact consist of discrete blocks of reliable data: if research interests or collection methods shift between observation periods, the quality of data regarding agonistic interactions may vary. Blocks with high-quality data interspersed with less reliable data may each display a burn-in period during which Elo-ratings track back to more accurate values. If this is not detected, it will undermine any determination of dominance hierarchies, ranks, and trajectories from such data sets, with these burn-in periods being mistaken for real shifts in relative dominance ranks. Similar issues may exist if there are disruptions to data collection, whether inadvertent or scheduled. In such situations, it may be better to reduce previously determined Elo-ratings to ordinal rankings rather than assuming accuracy in Elo-ratings that may be spurious.

Allowing *K* to vary with the intensity of aggression also improved the Elo-rating models in this study. The models including both prior history and varying *K* had only a single inconsistency with the pant-grunt data, which may have been the result of relative undersampling of an infrequently observed adolescent. Although varying *K* has been attempted before (Franz *et al.* 2015), that study used an indirect method of determining the intensity of aggression, in contrast to the directly observed intensities used here. The most important consequence of varying *K* is that it makes explicit the often-implicit assumptions concerning the relative importance of different intensities of aggression in altering dominance rank. When combining different types of intensities of aggression, as is typical when assessing social dominance in chimpanzees (Boesch and Boesch-Achermann 2000; Foster *et al.* 2009; Machanda *et al.* 2014; Muller 2002; Newton-Fisher 2002b; Nishida and Hosaka 1996; Watts 2000), these are rarely distinguished, even though a serious fight may have a much greater influence of subsequent dominance trajectories than a charging display, or a displacement (supplant) at a feeding site. In recent work using dominance ranks from the data set analyzed here, as well as observations from M-group in the Mahale Mountains National Park, Tanzania, Elo-ratings have been used successfully by employing the modified function presented here to minimize the burn-in period and explicitly incorporate varying intensities of aggression (Kaburu 2013; Kaburu and Newton-Fisher 2013, 2015a, b, 2016; Kaburu *et al*. 2013). Varying *K* at the level of the individual interaction also allows the effective intensity of aggression, i.e., its impact on dominance ranks, to be adjusted to accommodate context. Although for simplicity I assigned identical *K* values to behaviorally equivalent acts, it would be quite possible to model the effect of differing contexts: for example, should aggression during feeding have a greater or lesser effect than that occurring during resting? In the former, there may be a direct contest over resources, whereas in the latter, unpredictability may heighten the impact of any aggressive encounters. Similarly, aggression against a vulnerable individual such as a newly parous female, or an individual suffering from illness (Barrett 2016), may have greater impact on rank trajectories than interactions at other times or in other contexts. Choosing different *K* values, and using the Elo-rating method to model the impact, should allow such questions to be addressed.

Recent work by Foerster *et al*. (2016) takes a different approach to the variable *K*, determining an optimal value across interactions using maximum likelihood. Although this represents an improvement on an arbitrary value for *K*, and might be a useful method for determining rank across large time windows, employing any single value for *K* will inevitably smooth out the influence of intensity, and perhaps context, of interactions on future hierarchy position, i.e., Elo-ratings. In consequence, short-term hierarchy dynamics may be obscured, and hypotheses regarding intensity or context cannot be tested. An ideal way forward may be to combine approaches to *K*, in order to determine objectively the optimal values for each level of intensity.

The ability to adjust *K* on a per-interaction basis is also likely to be important when combining categorically distinct behaviors. In some studies, all win–loss interactions are considered when determining dominance rank, even when such interactions are not merely qualitatively different, but categorically so. This can be seen in many studies of wild chimpanzees where two categories of interactions, specifically those involving aggression and those involving pant-grunt vocalizations, are combined into a single measure of dominance (Boesch and Boesch-Achermann 2000; Foster *et al.* 2009; Machanda *et al.* 2014; Muehlenbein and Watts 2010; Newton-Fisher 2004; Nishida and Hosaka 1996; Watts 2000). However, Elo-ratings are typically determined using wins and losses from contests, with the resulting hierarchy based on agonistic dominance (Bernstein 1981; Maslow 1937; Mason 1983; Walters and Seyfarth 1987). In some recent studies (Fedurek *et al*. 2015b; Foerster *et al.* 2016), Elo ratings have been calculated from a combined data set of aggressive interactions and subordinate-initiated interactions, i.e., pant-grunting. Whether and to what extent this is appropriate remains an open question. The Elo-rating method scores each interaction as a win, loss, or draw, and alters the rank trajectory of both participants accordingly, weighting the impact of the win or loss by an expected probability of winning derived from the Elo-ratings of the participants before the interaction. It does not distinguish between categorically distinct behaviors. Thus a win (or loss) from a prolonged fight would have the same impact on Elo-ratings and rank trajectory as the receipt of a pant-grunt vocalization, if these were simply combined as win–loss interactions. The quite explicit assumption here is that receiving pant-grunts increases, while performing pant-grunts decreases, future hierarchy position, in an equivalent manner to an aggressive interaction. Whether this assumption is appropriate, whether pant-grunting (or similar behavior in other species) contributes but at a different level, or whether this vocalization merely functions as a confirmatory signal of current dominance relationships, remains undetermined. If pant-grunt interactions are to be considered in win–loss terms in the same model as aggressive interactions, it would be sensible to afford them different *K* values. While the social salience of pant-grunts (or equivalent signals of subordination in other species) suggests that they might be accorded high *K* values, if these vocalizations are largely confirmatory then *K* values should be substantially lower than those for aggressive interactions. If pant-grunts solely signal that an individual is subordinate to the recipient, and have no impact on rank trajectory (*K* = 0), then they are perhaps not suitable for the Elo-rating approach despite their usefulness in other methods. In more general terms, and across species, varying the value of *K* to account for both differing intensities and different categories of interaction (even if this value is zero) makes explicit their contributions to any model of dominance hierarchy.

Finally, the modifications proposed here—incorporating prior history and intensity of aggression—led to meaningful differences in the modeled hierarchy. Under the default approach, I found only a moderate relationship between dominance rank and mating success at best. By contrast, incorporating both prior history and intensity of aggression resulted in a hierarchy with a clear and substantial relationship between mating success and rank. This was true for both Elo-ratings and derived ordinal ranks. The position of an individual in the social hierarchy, its rank (ordinal or cardinal), is a key variable in the analysis of social behavior, whether analyzed directly, used as proxy for ability to control resources, or as a control. The results of this study show that assumptions made when determining dominance ranks can have important consequences for the interpretation of subsequent analyses. Embracing Elo-ratings offers the promise of a more accurate understanding of hierarchy dynamics and the influence of social rank, but we need to be careful that we understand the scale on which we are measuring, and how this is influenced by assumptions we make in its calculation.

In summary, the Elo-rating method is powerful and increasingly popular, but the existence of the burn-in period seriously hampers its usefulness. Incorporating even limited prior knowledge of dominance ranks substantially reduces this problem, and is likely to be of particular importance for relatively short studies, such as PhD research where subjects have been studied previously, and for disrupted longer-term data sets. Allowing the impact of different intensities of interaction to vary, i.e., adjusting the *K* value in the model, requires the often-implicit assumptions regarding the influence of different types of interactions on dominance rank to be made explicit. This can add clarity to reported research, improving cross-study comparisons. Having explicit control of the variable *K* at the level of the interaction also allows the Elo-rating method to be used to model the impact of differing assumptions, and to test specific hypotheses regarding the relative importance of different intensities and categories of interaction in generating social dominance hierarchies.

## Electronic Supplementary Material

Below is the link to the electronic supplementary material.

ESM 1 (DOCX 2237 kb)

ESM 2 (DOCX 122 kb)
