# Supplementary material for: Modeling Social Dominance: Elo-Ratings, Prior History, and the Intensity of Aggression
Source: Int J Primatol. 2017 Mar 16;38(3):427–47. doi: 10.1007/s10764-017-9952-2 (PMC5487812; doi:10.1007/s10764-017-9952-2)
Supplement: Supplementary file 1 — (DOCX 2237 kb) [file 10764_2017_9952_MOESM1_ESM.docx]

**Electronic Supplementary Material**

**Modeling Social Dominance: Elo-Ratings, Prior History, and the Intensity of Aggression**

**Nicholas E. Newton-Fisher**

**
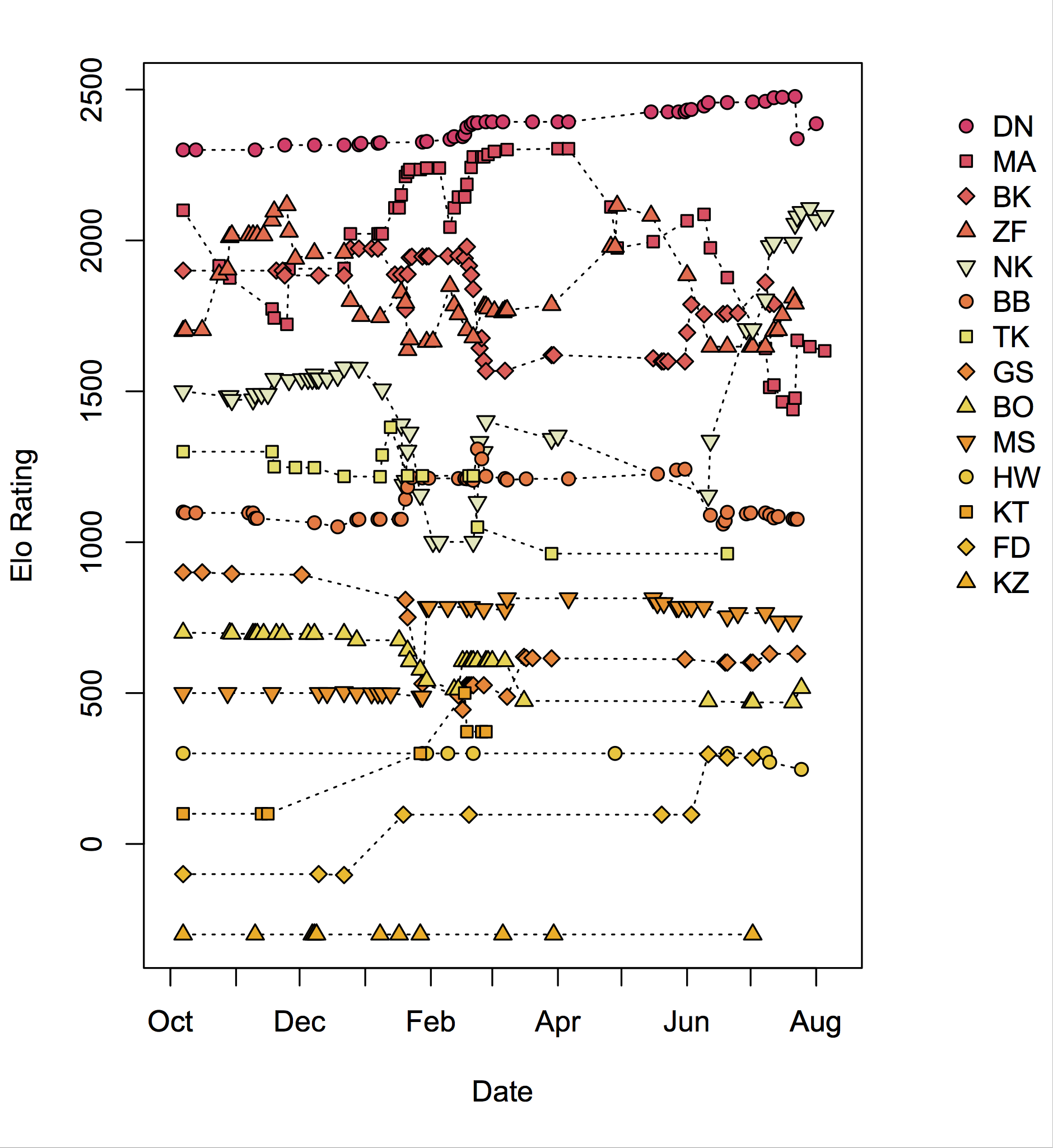
**

**Fig. S1** Rank trajectories for the adult and adolescent male chimpanzees (*Pan troglodytes*) of the Sonso community (Budongo, Uganda) between October 2003 and August 2004, as determined by an Elo-rating model that assigns starting according to prior records of dominance ranks.

**
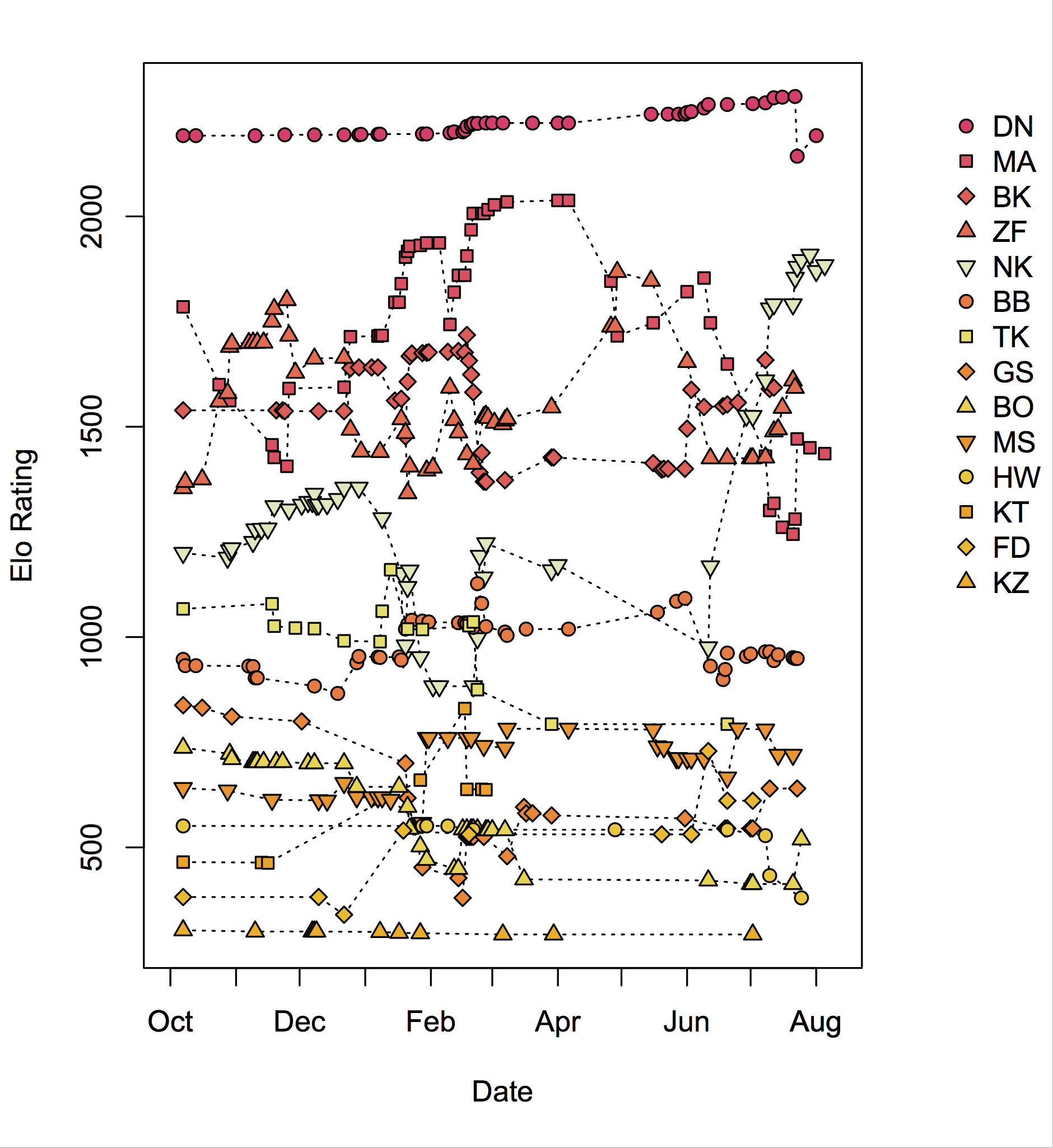
**

**Fig. S2** Rank trajectories for the adult and adolescent male chimpanzees (*Pan troglodytes*) of the Sonso community (Budongo, Uganda) between October 2003 and August 2004, as determined by an Elo-rating model that assigns starting Elo-ratings according to prior records of dominance ranks, applied using a negative exponential function.

**
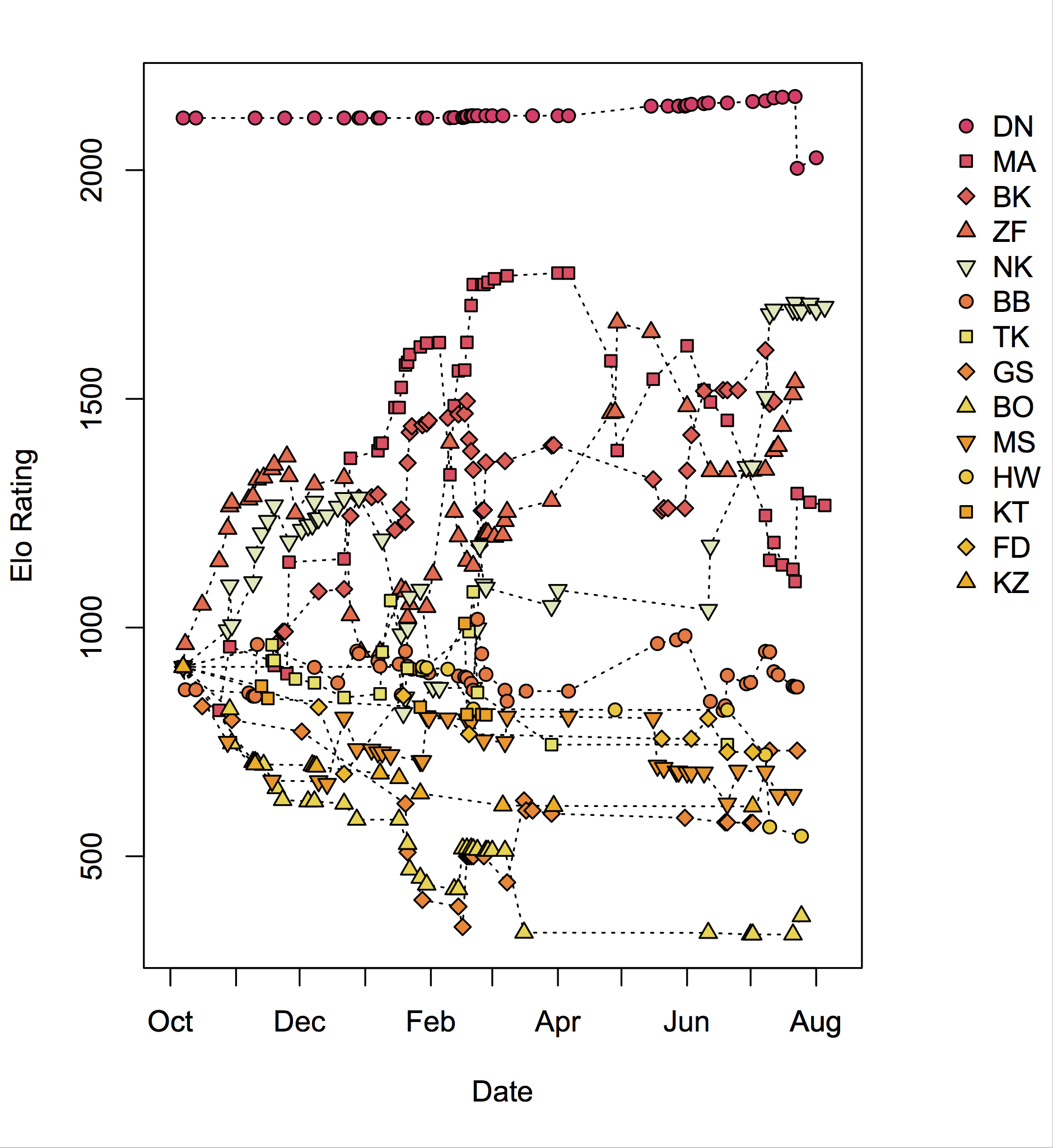
**

**Fig. S3** Rank trajectories for the adult and adolescent male chimpanzees (*Pan troglodytes*) of the Sonso community (Budongo, Uganda) between October 2003 and August 2004, as determined by an Elo-rating model that assigns a starting Elo-rating for the alpha male using a negative exponential function, but starts all other males at the same (default) Elo-rating on the assumption that their prior ranks are unknown.
