# Supplementary material for: Modeling Social Dominance: Elo-Ratings, Prior History, and the Intensity of Aggression
Source: Int J Primatol. 2017 Mar 16;38(3):427–47. doi: 10.1007/s10764-017-9952-2 (PMC5487812; doi:10.1007/s10764-017-9952-2)
Supplement: Supplementary file 2 — (DOCX 122 kb) [file 10764_2017_9952_MOESM2_ESM.docx]

**Electronic Supplementary Material**

**Modeling Social Dominance: Elo-Ratings, Prior History, and the Intensity of Aggression**

**Nicholas E. Newton-Fisher**

**Appendix:** R script for the revised *elo.sequence* function. Running this script in R will generate the function, and it should be used to overwrite the equivalent script from Neumann et al. (2011).

elo.sequence <-

function(datafile = "c:\\example data.csv", sep=",", startingvalue = 1000, constant_k = 200, priorElo = list(), priorRanks = list(), priorRankCategory = list(), priorRankIndex = 0, outcome = 1)

{

# 'datafile' : the path and file name of the csv data file with the interaction data; alternatively, will read existing dataframe

# 'startingvalue' : the amount of points each individual has at the beginning of the rating process (default is 1000, see example in Albers and de Vries, 2001)

# 'constant_k' : specify the amount your constant 'k' should take (default is 200, see example in Albers and de Vries, 2001)

# 'priorElo' : a list of IDs (case-sensitive), and associated initial Elo-rating values (e.g. ID1 = 2200, ID2 = 1500).

# 'priorRanks' : a list of IDs (case-sensitive), and associated starting ordinal ranks (e.g. ID1 = 1, ID2 = 2). Processed only if 'priorElo' not specified

# 'priorRankCategory' : a list of IDs (case sensitive), and associate rank categories ("alpha", high", "medium", "low"): will accept initial letters & is case insensitive (e.g. "a", "H" ...). Individuals not listed will start with default Elo-rating (i.e. 'startingvalue'). Processed only if 'priorElo' & 'priorRanks' not specified.

# ‘priorRankIndex’ : default is 0 for a distance between starting ranks equal to ‘k’; for a degree of despotism, such that elo-rating distances between high ranking males and others are exaggerated, enter reciprocal power: 0.3 works well.

# 'outcome' : 1 = first individual wins; 2 = second individual wins; 0 = draw

# reads the data file into the R workspace, or uses existing dataframe

if (is.data.frame(datafile)) IA_data <- datafile else IA_data <- read.csv(datafile, sep = sep)

# sets date variable

IA_data[, 1] <- as.Date(IA_data[, 1])

# assembles all individuals that are present in the data sheet

all_ids <- unique(c(as.character(IA_data$Winner),as.character(IA_data$Loser)))

# the default starting value of the Elo rating process (e.g. 1000)

currentELO <- rep(startingvalue,length(all_ids)); names(currentELO) <- all_ids

priorRanking = FALSE

# assigns starting Elo-ratings (user supplied)

if (length(priorElo) != 0)

{

rank_table = data.frame(subject = names(priorElo), elo = unlist(priorElo))

priorRanking = TRUE

}

# or calculates starting Elo-ratings from ordinal ranks (estimated using K difference between ranks)

else if (length(priorRanks) != 0)

{

# create table to hold subjects & ranks

rank_table = data.frame(subject = names(priorRanks), rank = unlist(priorRanks), elo = 0)

priorRanking = TRUE

# calculate starting Elo-ratings from ranks

avgrank <- with(rank_table, median(rank [rank!=0]))

for(i in 1:length(rank_table[,1]))

{

if(rank_table$rank[i] == 0) rank_table$elo[i] <- 0

else

{

startRank <- rank_table$rank[i]

recipRank <- startRank ^ (priorRankIndex * -1)

startingElo = startingvalue + ((avgrank - startRank) * (constant_k * recipRank))

rank_table[i, 3] <- startingElo

}

}

}

# or calculates starting Elo-ratings from ordered categorical ranks (high, medium, low)

else if (length(priorRankCategory) != 0)

{

# create table to hold subjects & ranks

rank_table = data.frame(subject = names(priorRankCategory), rankCat = unlist(priorRankCategory), rank = 0, elo = 0)

rank_table$rankCat = tolower(as.character(rank_table$rankCat))

priorRanking = TRUE

for(i in 1:length(all_ids)) # add all missing subjects, and assign to 'medium' rank category (results in 'startingvalue' Elo-rating)

{

if (!(all_ids[i] %in% rank_table$subject))

{

rank_table <- rbind(rank_table, data.frame(subject = all_ids[i], rankCat = "medium", rank = 0, elo = 0))

}

}

# assign an ordinal rank according to rank level

for(i in 1:length(rank_table$subject))

{

r <- substr(rank_table$rankCat[i], 1, 1)

if(identical(r, "a")) rank_table$rank[i] <- 1

if(identical(r, "h")) rank_table$rank[i] <- length(all_ids)/4

if(identical(r, "m")) rank_table$rank[i] <- length(all_ids)/2

if(identical(r, "l")) rank_table$rank[i] <- length(all_ids)-(length(all_ids)/4)

}

# calculate starting Elo-ratings from ordinal rank categories

avgrank <- with(rank_table, median(rank [rank!=0]))

for(i in 1:length(rank_table[,1]))

{

if(rank_table$rank[i] == 0) rank_table$elo[i] <- 0

else

{

startRank <- rank_table$rank[i]

recipRank <- startRank ^ (priorRankIndex * -1)

startingElo = startingvalue + ((avgrank - startRank) * (constant_k * recipRank))

rank_table[i, 4] <- startingElo

}

}

}

if (priorRanking == TRUE)

{

if (length(priorElo) == 0)

{

# centre calculated Elo-ratings so that their average matches the specified starting value

meanElo <- with(rank_table, mean(elo [elo!=0]))

for(i in 1:length(all_ids)) rank_table$elo[i] = round(rank_table$elo[i] - (meanElo - startingvalue))

}

# assign prior Elo-ratings to 'currentELO'

for(i in 1:length(all_ids))

{

if (is.null(rank_table$elo[i])) startValue <- startingvalue

else if(is.na(rank_table$elo[i])) startValue <- startingvalue

else startValue <- rank_table$elo[i]

subject <- as.character(rank_table$subject[i])

startValue -> currentELO[which(names(currentELO)==subject)]

}

}

# creates the table in which the new ratings after each encounter are saved (the first values equal the initial values that were defined in the step above)

log_table <- as.data.frame(matrix(ncol=6, nrow=(length(IA_data[, 1]) * 2 + length(all_ids))))

colnames(log_table) <- c("IA_no", "ID", "Date", "elo", "K", "Outcome")

log_table[, 2] <- as.character(log_table[, 2])

# the first (starting) values are now filled into first rows of the table (depending on the number of individuals)

log_table[1:length(all_ids), 1] <- 0 # interaction no '0'

log_table[1:length(all_ids), 2] <- as.character(all_ids) # all IDs

rankDate <- IA_data[1, 1]-1 # assign a starting date for pre-existing ranks

log_table[1:length(all_ids), 3] <- rankDate #

class(log_table$Date) <- "Date" # formatting the Date column

log_table[1:length(all_ids), 4] <- currentELO # the starting value of the Elo rating process (e.g. 1000)

log_table[1:length(all_ids), 5] <- constant_k # the default constant_k value

log_table[1:length(all_ids), 6] <- outcome # the default outcome value

# this is the actual loop that calculates the new elo ratings after each interaction and adds them into the 'log_table', and also keeps the 'currentELO' table updated

for(i in 1:length(IA_data[,1]))

{

# makes sure the IDs are formatted as character strings

cont1 <- as.character(IA_data$Winner[i])

cont2 <- as.character(IA_data$Loser[i])

# retrieves relevant constant_k value; uses default if absent

if(is.null(IA_data$K[i])) constK = constant_k

else if(is.na(IA_data$K[i])) constK = constant_k

else constK <- as.numeric(IA_data$K[i])

# display constant_k value used

log_table[(length(all_ids) + (2 * i - 1)):(length(all_ids) + (2 * i)), 5] <- constK

# retrieves relevant outcome value; uses default if absent

if(is.null(IA_data$Outcome[i])) outCm = outcome

else if(is.na(IA_data$K[i])) outCm = outcome

else outCm <- as.numeric(IA_data$Outcome[i])

# display outcome value used

log_table[(length(all_ids) + (2 * i - 1)):(length(all_ids) + (2 * i)), 6] <- outCm

# calculates the new ratings for the two interacting individuals

log_table[(length(all_ids) + (2 * i - 1)):(length(all_ids) + (2 * i)), 4] <- as.numeric(elo.single(currentELO[which(names(currentELO)==cont1)], currentELO[which(names(currentELO)==cont2)], outcome=outCm, constant_k=constK))

# fills the respective date of the interaction

log_table[(length(all_ids) + (2 * i - 1)):(length(all_ids) + (2 * i)), 3] <- as.Date(IA_data$Date[i])

# fills the IDs of the two interacting individuals

log_table[(length(all_ids) + (2 * i - 1)):(length(all_ids) + (2 * i)), 2] <- c(cont1, cont2)

# count of the interaction

log_table[(length(all_ids) + (2 * i - 1)):(length(all_ids) + (2 * i)), 1] <- i

# updates the 'currentELO' table with the above calculated ratings of the two interacting individuals

currentELO[which(names(currentELO)==cont1)] <- log_table[(length(all_ids) + (2 * i - 1)), 4]

currentELO[which(names(currentELO)==cont2)] <- log_table[(length(all_ids) + (2 * i)), 4]

} # end of the loop

# returns a table with all ratings

return(log_table)

}
